# Restriction of extracellular lipids renders pancreatic cancer dependent on autophagy

**DOI:** 10.1186/s13046-021-02231-y

**Published:** 2022-01-08

**Authors:** Maria Saliakoura, Matteo Rossi Sebastiano, Ioanna Nikdima, Chiara Pozzato, Georgia Konstantinidou

**Affiliations:** Institute of Pharmacology, University of Bern, 3010 Bern, Switzerland

**Keywords:** Pancreatic cancer, Lipid metabolism, Extracellular lipids, Combination therapy, Tumor metabolic vulnerabilities

## Abstract

**Background:**

KRAS is the predominant oncogene mutated in pancreatic ductal adenocarcinoma (PDAC), the fourth cause of cancer-related deaths worldwide. Mutant KRAS-driven tumors are metabolically programmed to support their growth and survival, which can be used to identify metabolic vulnerabilities. In the present study, we aimed to understand the role of extracellularly derived fatty acids in KRAS-driven pancreatic cancer.

**Methods:**

To assess the dependence of PDAC cells on extracellular fatty acids we employed delipidated serum or RNAi-mediated suppression of ACSL3 (to inhibit the activation and cellular retention of extracellular fatty acids) followed by cell proliferation assays, qPCR, apoptosis assays, immunoblots and fluorescence microscopy experiments. To assess autophagy in vivo, we employed the *Kras*^*G12D/+*^;*p53*^*flox/flox*^;*Pdx1-*Cre^ERT2^ (KPC) mice crossed with *Acsl3* knockout mice, and to assess the efficacy of the combination therapy of ACSL3 and autophagy inhibition we used xenografted human cancer cell-derived tumors in immunocompromised mice.

**Results:**

Here we show that depletion of extracellularly derived lipids either by serum lipid restriction or suppression of ACSL3, triggers autophagy, a process that protects PDAC cells from the reduction of bioenergetic intermediates. Combined extracellular lipid deprivation and autophagy inhibition exhibits anti-proliferative and pro-apoptotic effects against PDAC cell lines in vitro and promotes suppression of xenografted human pancreatic cancer cell-derived tumors in mice. Therefore, we propose lipid deprivation and autophagy blockade as a potential co-targeting strategy for PDAC treatment.

**Conclusions:**

Our work unravels a central role of extracellular lipid supply in ensuring fatty acid provision in cancer cells, unmasking a previously unappreciated metabolic vulnerability of PDAC cells.

**Supplementary Information:**

The online version contains supplementary material available at 10.1186/s13046-021-02231-y.

## Background

KRAS is a critical driver of pancreatic ductal adenocarcinoma (PDAC) initiation and maintenance [1]. The 5-year survival of PDAC patients is ~ 10%, occurring mainly due to late diagnosis, therapy resistance and rapid recurrence [2, 3]. Although approved targeted therapies are now available for some KRAS-driven tumors such as lung and colorectal cancer, no effective targeted therapies have been found for PDAC [4]. Therefore, new therapeutic approaches for pancreatic cancer are urgently needed.

In order to ensure unconstrained proliferation and metabolic flexibility, KRAS-mutant tumors put in place several different metabolic adaptations, allowing them to scavenge or recycle various building blocks and metabolites [5]. Ultimately, these metabolic adaptations are critical for the maintenance of these tumors and their identification may provide therapeutic opportunities.

One known metabolic adaptation of KRAS-driven tumors is the scavenging of extracellular unsaturated fatty acids (FAs) [6]. We recently showed that the acyl-coA synthetase long chain 3 (ACSL3), an enzyme that promotes the activation and retention of extracellular unsaturated FAs by converting them into hydrophilic fatty acyl-CoA esters that cannot exit cells [7, 8], is overexpressed in KRAS-driven lung and pancreatic cancer [9, 10]. In lung cancer, ACSL3 plays an essential role in mediating the activation and channeling of extracellularly derived FAs into mitochondria for fatty acid oxidation and prostaglandin synthesis [9, 11]. In mouse pancreatic cancer, *Acsl3* deletion triggered reduced tumor cell proliferation, in part by impairing the production of the pro-fibrotic plasminogen activator inhibitor-1 (PAI-1), decreasing desmoplasia and immunosuppression [10]. However, the functional role of the extracellularly derived lipids in pancreatic cancer cells remains unknown.

In the present study, we aimed to understand the role of extracellularly derived FAs in KRAS-driven pancreatic cancer. By combining in vitro cell-based assays and genetic manipulations in both cancer cells and mouse models we show that restriction of extracellularly derived FAs reduces pancreatic cancer cell proliferation, while differentially affects pancreatic cancer cell survival. We found that the restriction of extracellularly derived FAs triggers a compensatory increase in autophagic flux that can be exploited as a cancer dependency. Accordingly, pharmacological inhibition of autophagy enhanced extracellular lipid restriction-mediated induction of cell death and inhibited PDAC growth. Therefore, we have exposed a new metabolic vulnerability of PDAC tumors on autophagy that could be exploited for therapeutic purposes.

## Methods

### Cell lines

The HPDE-i*KRAS*^G12D^ cell line was kindly provided by Prof. Kenneth L. Scott [12], and cultured in KSFM (Gibco) supplemented with EGF, Bovine Pituitary extract (Gibco). For the experiments, HPDE cells were cultured in RPMI 1640 (Gibco) supplied with 10% FBS and Penicillin/Streptomycin for at least 2 passages and, when indicated, expression of KRAS^G12D^ (HPDEK) was induced by addition of 500 ng/ml doxycycline (Fisher Scientific, BP2653) in the culture medium for 72 h. AsPC-1 and SU68.86 were cultured in RPMI-1640 (Gibco), MiaPaCa-2, Hs766S, CFPAC-1 and PANC-1 cell lines were cultured in DMEM (Gibco); media were supplied with 10% FBS (Thermo Fisher) or delipidated FBS (via fumed silica precipitation method; Biowest #S181L). All cell lines were DNA fingerprinted for provenance, screened for mycoplasma and cultured in an incubator at 37 °C and 5% CO_2_.

### shRNAs, virus production and transduction

The validated shRNAs were obtained as bacterial glycerol stock from Sigma Aldrich and have been previously tested [10, 11]. Recombinant lentiviruses were produced by transfecting HEK 293 T cells, using the *Trans*IT®-293 Transfection Reagent (Mirus; MIR2705), with pCMV-VSV-G (VSV-G protein), pCMV-dR8.2 (lentivirus packaging vector) and lentiviral constructs, according to the manufacturer’s instructions. For lentiviral production pLKO.1 puro (Addgene plasmid #8453; http://n2t.net/addgene:8453; RRID:Addgene_8453), pCMV-VSV-G (Addgene plasmid #8454; http://n2t.net/addgene:8454; RRID:Addgene_8454) and pCMV-dR8.2 dvpr (Addgene plasmid #8455; http://n2t.net/addgene:8455; RRID:Addgene_8455) were used and they were a gift from Prof. Bob Weinberg. The full list of the shRNA sequences used in this manuscript can be found in the Supplemental Reagents Table 1.

### Animal studies

The mixed background, tamoxifen inducible *LSL-Kras*^*G12D/+*^*;Trp53*^*flfl*^;*Pdx1-Cre*^*ERT2*^*;Acsl3*^*−/−*^ mouse model was obtained by crossing the strain Tg (Pdx1-cre/Esr1*)#Dam/J (from JaxLab, Stock number 024968) with B6.129SS4-kras^tm4Tyj^/J (from JaxLab, Stock number 008179), B6.129P2-Trp53^tm1Brn^/J (from JaxLab, Stock number 008462) and LEXKO 011 (Constitutive *Acsl3*^*−/−*^ mouse, obtained from the European Mouse Mutant Archive). Mice were backcrossed for twelve generations, before creating the experimental groups. The xenograft PDAC mouse model was performed on NOD.Cg-Prkdc^scid^ Il2rg^tm1Wjl^/SzJ (NSG) immunodeficient mice.

Mice were maintained under controlled humidity and temperature conditions, with a standard 12 h light/dark cycle and were fed ad libitum. Genomic DNA extraction and PCR assay were performed using the KAPA HotStart Mouse Genotyping Kit (Kapa Biosystems, KK7352) and KAPA2G Fast HotStart Genotyping Mix (Kapa Biosystems, KK5621) respectively, according to the manufacturer’s instructions. The mice genotypes were confirmed following the corresponding JaxLab protocols. The PCR for the *Acsl3* genotyping was performed according to the KAPA HotStart Mouse Genotyping Kit with an annealing temperature at 60 °C. The full list of oligos used to genotype the mice can be found in the Supplemental Reagents Table 1.

Recombination in *LSL-Kras*^*G12D/+*^*;Trp53*^*flfl*^*;Pdx1-Cre*^*ERT2*^ mice was induced at 3 weeks of age with five subsequent daily administrations of tamoxifen by intraperitoneal injection. The injected mixture contained tamoxifen pre-solubilized in ethanol and diluted in sunflower oil (Sigma Aldrich) with a ratio of 1:9 parts ethanol/sunflower oil. 2.5 mg of tamoxifen were administered to each mouse with each injection. *LSL-Kras*^*G12D/+*^*;Trp53*^*fl/fl*^*;Pdx1-Cre*^*ERT2*^ mice were sacrificed 8 weeks post induction (11 weeks of age). Pancreata were retrieved after anesthesia and perfusion of the animals with PBS.

For the xenograft PDAC mouse model, 2 × 10^6^ cells resuspended in 100 μl PBS were injected subcutaneously in the right upper hind limb of male NSG mice. Visual check was performed daily and caliper measurement of the growing tumor was performed every second day. HCQ was solubilized in water in a stock concentration and diluted in PBS at a dose of 60 mg/kg in a volume of 100 μl. HCQ was intraperitoneally administrated thrice, on the 20th, 22nd and 24th day after cell injection.

### RT- PCR

RNA was extracted using the RNAeasy kit, QIAGEN, 74104) and cDNA was synthesized with the RevertAid First Strand cDNA Synthesis Kit (Thermo Scientific, K1622). qPCR was performed in 96 well plates (TreffLab) with FastSybr green (Thermo Scientific, 4,367,659). The normalization was performed with the ΔΔCT method. The full list of the oligonucleotides can be found in the Supplemental Reagents Table 1.

### Histology

Tissues for histology were fixed at 4 °C overnight in 4% paraformaldehyde before paraffin embedding. All sections used for histological analysis were 5 μm thick.

### Immunofluorescence

For immunofluorescence (IF) conducted on paraffin-embedded mouse pancreatic cancer tissue all stainings with primary antibodies were performed as previously described [13]. Briefly, after deparaffinization, tissue was rehydrated through a graded series of alcohol, followed by boiling for 15 min in Sodium Citrate buffer (pH 6) and blocking in 2% BSA in PBS containing 0.1% Tween. Antibodies were diluted in blocking solution and incubation was done at 4 °C overnight. Secondary fluorescent-tagged antibodies were from molecular probes (Invitrogen). Stained cells and sections images were acquired with a confocal microscope (Carl Zeiss). Image analysis was performed with Imaris software.

### Immunoblotting

Cells were lysed in RIPA buffer (50 mM Tris-HCl pH 8.0, 150 mM NaCl, 1.0% NP-40, 0.5% sodium deoxycholate, 0.1% SDS) containing complete EDTA-free protease inhibitors (Roche) and 1 mM PMSF. Mouse samples were pulverized in a liquid nitrogen mortar and homogenized in RIPA buffer, prior to lysis incubation. Samples were resolved by SDS-PAGE in Bio-Rad blotting chamber, transferred to nitrocellulose membrane using a semi-dry chamber (Bio Rad) and blocked in 5% BSA in PBS containing 0.1% Tween. Membranes were then incubated overnight at 4 °C with primary antibody diluted in blocking buffer. Secondary fluorescent-tagged antibodies were from Li-Cor biosciences and development was done in Li-Cor fluorescence-chemiluminescence detector. All antibodies and their dilutions are listed in the Supplemental Reagents Table 1.

### Cell proliferation assay

Cells were plated in 24-wells plates (8 × 10^3^ cells/well) and let proliferate for 48 or 96 h. Cell viability was measured by crystal violet (Sigma Aldrich) staining (0.1%) of adherent cells after 10 min fixation in 4% paraformaldehyde (Sigma Aldrich). After washing and air-drying, stained cells were de-coloured with 10% acetic acid and OD600 was measured with a spectrophotometer.

### Annexin/PI measurement by flow cytometry

For flow cytometry analysis, cells were gated according to their forward and side scatter values to exclude cell clusters. Cells were plated in 12-well plates (4 × 10^4^ cells/well) and treated as described in the figure legends. On the day of the assay, cells were collected, washed with staining buffer (150 mM NaCl, 4 mM KCl, 2.5 mM CaCl_2_, 1 mM MgSO_4_, 15 mM HEPES pH 7.2, 2% FBS and 10 mM NaN_3_) and stained with Atto633-conjugated Annexin V for 20 min in the dark, on ice. Cells were then washed with staining buffer and resuspended in 200 μl PI at a final concentration of 2 μg/mL. Flow cytometry was performed following a standard procedure with a FACS Lyric instrument (BD Biosciences). Data were analysed using the FlowJo V10 workspace.

### Caspase-3 activity assay

Caspase-3 activation was detected with the EnzCheck® Caspase-3 Assay kit #2 (Thermo Fisher, catalog n. E6646) according to the manufacturer’s instructions.

### Lipid droplets measurement by flow cytometry

For LD measurement, 1 × 10^6^ cells were gently washed with PBS and fixed with 4% paraformaldehyde for 10 min at room temperature. After removing the fixative solution, cells were washed 3 times with PBS to remove residual paraformaldehyde and stained with LipidTOX Deep Red Neutral Lipid (Invitrogen, H34477) for 30 min. The cells were then washed with PBS before performing the assay as previously described [14].

### Confocal fluorescence microscopy

For the BODIPY™ 500/510 C4, C9 staining, 2 × 10^4^ cells were seeded on glass coverslips in 24-well plates. 24 h later, the BODIPY™ 500/510 C4, C9 (Invitrogen, B3824) was added to the cell culture media for 30 min, cells were fixed with 4% paraformaldehyde for 10 min, washed with PBS and mounted for analysis.

The staining with BODIPY 493/503 (Invitrogen, D3922) was performed as previously described [15].

For Cherry-EGFP-LC3B imaging, cells were transduced as indicated in Fig. 3B and plated for imaging by confocal fluorescence microscopy or imaging flow cytometry.

### Imaging flow cytometry

The Imaging flow cytometry was performed with an Amnis ImageStream X®, model MK II from Luminex. The procedure started with automatic gating of forward and side scatter, followed by acquisition of 10^4^ events/condition in the 595–640 nm channel (mCherry), the 505–560 nm channel (GFP) and brightfield (as visual control). The machine was operated through the control software INSPIRE®. The analysis of the results was performed with the package IDEAS®, distributed as official analysis tool by Amnis. The analysis procedure consisted in following the SPOT® wizard, subsection deputed to the quantification of luminous spots within the frames, with standard parameters. The output results are in the form of average detected spots frequency, while the R1 threshold is automatically determined by the software and indicates the confidence threshold of the control condition.

### Fluorescence quantification and analysis

All fluorescence microscopy analyses were performed using the Imaris Studio Software (www.bitplane.com). In order to exclude false positivity due to the background of the green channel, a first mask was set on it, with a threshold value of “1” for bright intensity. The confocal IF analyses were performed with the Imaris Spots or Surface wizard. All results are the average of at least 15 pictures.

### Statistical analysis and elaboration

All data presented are expressed as mean ± s.d or s.e.m. of experiments repeated at least three times. We determined the significance of results by employing unpaired Student’s t test or one-way or two-way ANOVA, multiple comparison test by using GraphPad Prism vv.7.00 (GraphPad Software, San Diego, Ca).

## Results

### ACSL3 knockdown impairs extracellularly derived FAs activation and reduces PDAC cell proliferation

**Fig. 1:**
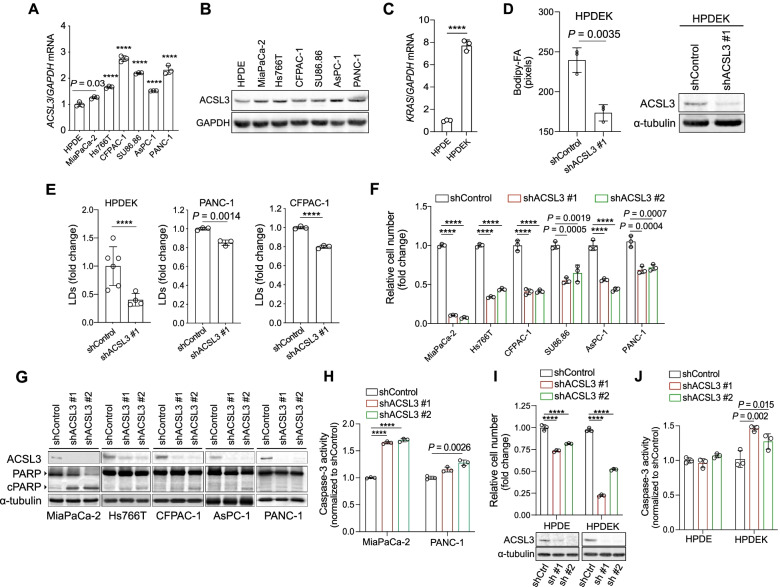
ACSL3 knockdown reduces the proliferation of PDAC cells. **A and B** *ACSL3* mRNA (A) or protein (B) level in immortalized HPDE and the indicated human pancreatic cancer cell lines. HPDE: human pancreatic ductal epithelial cells; *n* = 3. **C** *KRAS* mRNA level of HPDE cells treated with 500 ng/ml doxycycline for 72 h to induce *KRAS*^*G12D*^ (HPDEK); *n* = 3. **D** Bodipy-FA uptake of HPDEK cells transduced either with an empty vector (pLKO-puro, shControl) or a shRNA against ACSL3 (shACSL3 #1), stained with BODIPY 500/510 C4, C9 and analysed by confocal fluorescence microscopy (left), and immunoblot showing ACSL3 knockdown efficiency (right). Statistical analysis was performed from ~ 150 cells/sample; *n* = 3. **E** Lipid droplets (LDs) staining with LipidTOX followed by flow cytometry quantification of HPDEK (*n* = 4–6), PANC-1 (*n* = 3) and CFPAC-1 (*n* = 3) cells transduced as in (D). **F** Relative cell number of the indicated cell lines transduced either with an empty vector (pLKO-puro, shControl) or 2 different shRNAs against ACSL3; *n* = 3. **G** Immunoblot analysis of the indicated cell lines transduced as in (F). **H** Caspase-3 activity of MiaPaca-2 and PANC-1 cells transduced as in (F) before plating for the assay; *n* = 3. **I** Relative cell number (top) and immunoblot to show ACSL3 knockdown efficiency (bottom) of HPDE and HPDEK cells transduced either with an empty vector (pLKO-puro, shControl) or 2 different shRNAs against ACSL3 and treated with 500 ng/ml doxycycline (HPDEK) for 72 h before plating for the assay; shCtrl: shControl, sh #1: shACSL3 #1, sh #2: shACSL3 #2; *n* = 3. **J** Caspase-3 activity of HPDE cells transduced and treated as in (I); *n* = 3. Graphical data are shown as the mean ± s.d. Statistical analyses were done using two-tailed unpaired Student’s t-test or one-way ANOVA. **** *P* < 0.0001; n, number of biologically independent samples

ACSL3 is overexpressed in human and mouse pancreatic cancer tissue compared to healthy pancreas [10]. To find out to which extent the expression of ACSL3 in PDAC cell lines agrees with patient samples, we assessed the mRNA and protein levels of ACSL3 in a panel of 6 KRAS mutant human PDAC cell lines, namely MiaPaCa-2, Hs766T, CFPAC1, SU86.86, AsPC-1 and PANC-1. We found that all PDAC cell lines evidenced higher ACSL3 mRNA and protein levels compared to immortalized human pancreatic duct epithelial cells (HPDE), which derive from healthy pancreas (Fig. 1A and B) [16].

We previously provided evidence that ACSL3 mediates the activation and retention of extracellularly derived FAs in KRAS mutant lung cancer cells [9]. Accordingly, RNAi-mediated knockdown of ACSL3 in HPDE-i*KRAS*^G12D^ cells, which are engineered to carry a doxycycline–inducible oncogenic *KRAS*^*G12D*^ expression (thereafter named HPDEK) (Fig. 1C) [12], leads to a reduction of extracellularly derived FAs activation (Fig. 1D). This is evidenced by the reduced retention of extracellularly supplied FAs labelled with the fluorescent FA BODIPY 500/510 C4,9 (Bodipy-FA), which mimics a long-chain FA (Fig. 1D). Accordingly, staining of lipid droplets (LDs) with LipidTOX, a LD marker, and analysis by flow cytometry showed a reduction in the deposition of lipids in LDs upon ACSL3 knockdown in PDAC cell lines, confirming a decrease in lipid retention (Fig. 1E).

We then aimed to evaluate whether suppression of ACSL3 affects human pancreatic cancer cell proliferation. To this end, we knocked down ACSL3 with 2 different shRNAs and measured the relative cell number 72 h later (Fig. 1F). We found 50% reduction of relative cell number compared to the control in five out of six cell lines (Hs766T, CFPAC1, SU86.86, AsPC-1 and PANC-1) and a 90% reduction in MiaPaCa-2 cells. Moreover, immunoblot for poly ADP-ribose polymerase (PARP) revealed a marked increase in cleaved PARP, a marker of cell death, only in MiaPaCa-2 cells, while for all the other cell lines the increase was mild or undetectable (Fig. 1G). This disparate effect in cell death upon ACSL3 knockdown was also confirmed by measuring caspase-3 activity upon ACSL3 knockdown in 2 representative cell lines MiaPaCa-2 and PANC-1 (Fig. 1H).

In order to assess whether ACSL3 is an exclusive vulnerability of KRAS mutant cells, we performed cell proliferation assays in HPDE and HPDEK cells. Interestingly, ACSL3 knockdown in HPDE cells showed a modest reduction in cell number, while the number of *KRAS*^G12D^ expressing HPDEK cells was drastically reduced (Fig. 1I). The anti-proliferative effect in HPDEK cells was also associated with an increase in caspase-3 activity, while no effect was observed in HPDE cells (Fig. 1J).

Our results indicate that in pancreatic cancer cells ACSL3 loss-of-function impairs extracellularly derived FAs cellular retention, reduces cell proliferation and variably induces cell death.

### Restriction of extracellularly derived FAs recapitulates the impact of ACSL3 suppression on PDAC cell proliferation

**Fig. 2:**
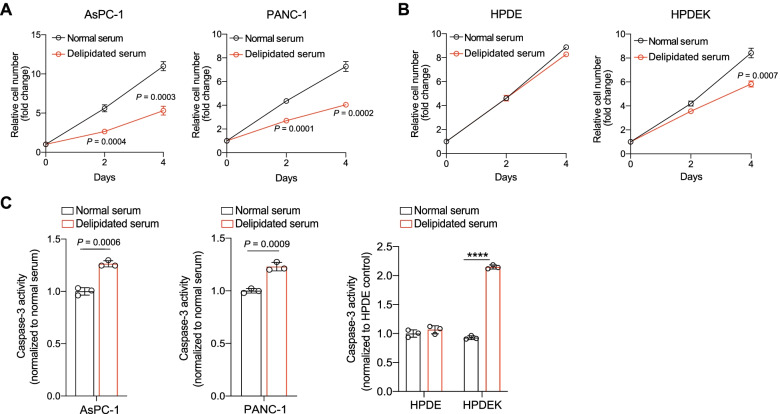
Extracellular lipid deprivation reduces the proliferation of PDAC cells. **A** Relative cell number of AsPC-1 and PANC-1 cells treated with media containing normal or delipidated serum; *n* = 3. **B** Relative cell number of HPDE and HPDEK cells treated as in (A); *n* = 3. **C** Caspase-3 activity of the indicated cell lines treated with media containing normal or delipidated serum for 72 h; *n* = 3. Graphical data are shown as the mean ± s.d. Statistical analyses were done using two-tailed unpaired Student’s t-test or one-way ANOVA; n, number of biologically independent samples

The reduced proliferation of PDAC cells upon ACSL3 suppression could be caused by an impaired cellular retention of extracellularly derived FAs. To directly test this possibility, we assessed the impact of extracellular lipid restriction in KRAS mutant cancer cells. For this purpose, we incubated PDAC cell lines with media containing normal or lipid depleted serum. We used 2 different human KRAS mutant PDAC cell lines, AsPC-1 and PANC-1, as well as HPDE and HPDEK cells. Similar to ACSL3 suppression, switch from normal to lipid depleted media caused a reduction in cell proliferation of AsPC-1, PANC-1 and HPDEK cells lines, but not of HPDE (lacking mutant *KRAS*) cells (Fig. 2A and B). Notably, serum lipid depletion did not affect the protein levels of ACSL3 or other ACSL isoenzymes, excluding the possibility that the reduced cell proliferation could be caused by changes in ACSL isoforms (Supplementary Fig. 1A and 1B).

Treatment of AsPC-1 and PANC-1 cells with media containing delipidated serum caused a mild increase in caspase-3 activity (1.2-fold) compared to the normal serum-treated cells (Fig. 2C). Furthermore, assessment of apoptosis by flow cytometry revealed no change in the percentage of Annexin V/propidium iodide (ANN/PI)-positive cells, confirming that lipid depletion is not sufficient to trigger a detectable apoptotic cell death (Supplementary Fig. 1C). Notably, the treatment with delipidated serum induced a 2-fold increase in caspase-3 activity of HPDEK cells (Fig. 2C), and a similar change in the percentage of ANN/PI-positive cells, indicating increased sensitivity to serum lipid depletion compared to PDAC cell lines (Supplementary Fig. 1D).

Taken together, these results suggest that the extracellular lipid depletion reduces cell proliferation in all KRAS mutant cancer cell lines, while causing only a moderate increase in cell death in PDAC cells.

### ACSL3 suppression or serum lipid depletion increase the autophagic flux of PDAC cells

**Fig. 3:**
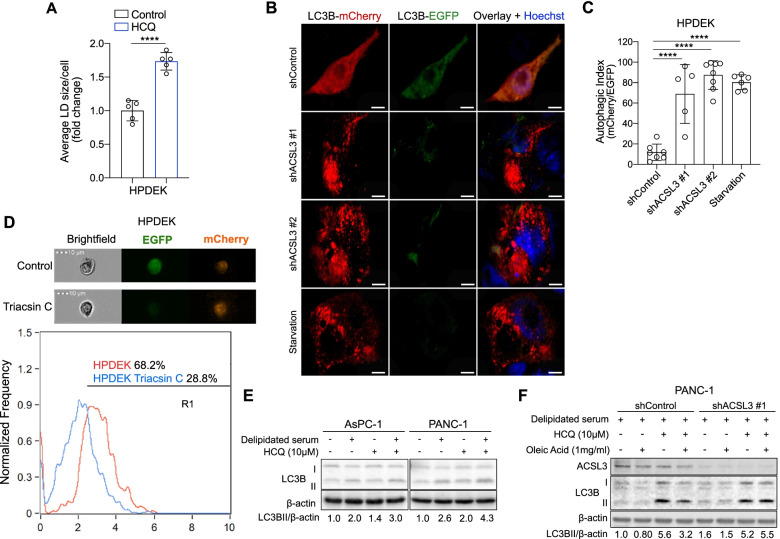
Extracellular lipid deprivation enhances autophagy in PDAC cells. **A** Average lipid droplet (LD) size of HPDEK cells stained with BODIPY 493/503. Cells were first treated with 10 μM HCQ for 4 h followed by staining with BODIPY 493/503 and confocal fluorescence microscopy analysis. Statistical analysis was performed from ~ 150 cells/sample. HCQ: hydroxychloroquine; *n* = 5. **B and C** Representative images of LC3B-mCherry and LC3B-EGFP (B) and autophagic index quantification (C) of HPDEK cells transduced either with an empty vector (pLKO-puro, shControl) or 2 different shRNAs against ACSL3. Overnight starvation was used as a positive control for autophagy induction; *n* = 5–8. **D** Representative images (top) and co-localization score plot between mCherry and EGFP signal from the imaging flow cytometer of HPDEK cells treated with 2.5 μM Triacsin C for 24 h. **E** Immunoblot analysis of AsPC-1 and PANC-1 cells treated with normal or delipidated serum for 72 h followed by treatment with 10 μM HCQ for 4 h, when indicated. HCQ: hydroxychloroquine. **F** Immunoblot analysis of PANC-1 cells transduced as indicated and treated with delipidated serum for 72 h followed by treatment with 10 μM HCQ for 4 h and/or 1 mg/ml of oleic acid for 3 h, when indicated. HCQ: hydroxychloroquine. Graphical data are shown as the mean ± s.d. Statistical analyses were done using one-way ANOVA. **** *P* < 0.0001; n, number of biologically independent samples

The lack of an obvious cell death induction in all PDAC cell lines tested upon ACSL3 suppression or serum lipid depletion suggests the insurgence of possible mechanisms that provide a source of FAs to replenish the missing lipids. A potential candidate mechanism that could recycle cellular components, in order to provide lipids, is autophagy [17, 18]. Indeed, there is strong evidence indicating that autophagy acts in regulating the turnover of lipids from intracellular lipid stores (macrolipophagy) and consequently, inhibition of autophagy increases lipid storage in lipid droplets (LDs) [17]. To assess whether the autophagic flux status impacts LDs turnover in pancreatic cancer cells, we inhibited autophagy with HCQ, an inhibitor of autophagosome–lysosomal fusion and measured the abundance of LDs by staining of HPDEK cells with BODIPY 493/503. We found that inhibition of autophagy increased the accumulation of lipids in LDs, suggesting that autophagy acts to maintain a high LD turnover in pancreatic cancer cells under basal conditions (Fig. 3A).

To evaluate whether ACSL3 suppression leads to an increase in autophagic flux, we knocked down ACSL3 with 2 different shRNAs in HPDEK cells and transduced them with a plasmid expressing mCherry-EGFP-LC3B, which is a tandem reporter for autophagosomes. Due to the low pH of lysosomes, the fluorescence of EGFP is quenched whereas the mCherry remains stable. Therefore, an increase in autophagic flux is manifested as quenching of the EGFP fluorescence and the autophagic index is determined by the mCherry/EGFP ratio [19, 20]. Confocal microscopy evidenced a significant induction of the autophagic index upon ACSL3 knockdown that was similar to serum starvation, the positive control for autophagy induction (Fig. 3B and C). Furthermore, imaging flow cytometry analysis upon treatment of mCherry-GFP-LC3B transduced HPDEK cells with Triacsin C, an ACSL3 inhibitor, revealed a reduction of EGFP-positive cells indicative of increased autophagy (Fig. 3D). These results were also confirmed by assessing the autophagy markers LC3B-II and p62. Specifically, immunoblot analysis revealed a reduction in p62 (indicative of increased p62 degradation) and an increase in LC3B-II upon ACSL3 knockdown, indicating increased autophagic flux (Supplementary Fig. 2A). Moreover, LC3B-II increased upon ACSL3 knockdown also in MiaPaCa-2 and PANC-1 cell lines (Supplementary Fig. 2B).

Next, we assessed the induction of autophagy upon serum lipid depletion. Immunoblot analysis evidenced a marked induction of autophagy in AsPC-1 and PANC-1 cell lines as shown by an increase in LC3B-II protein level (Fig. 3E). Since an increase in LC3B-II could also be caused by a block in autophagosomal maturation [21], we inhibited autophagy with HCQ, which blocks autophagosome–lysosomal fusion. In the presence of HCQ, serum lipid depletion caused a further increase in LC3B-II compared to the single treatments (serum lipid depletion alone and HCQ alone), confirming increased autophagic flux, rather than a block in autophagosomal maturation (Fig. 3E).

To prove that it is the depletion of extracellularly derived lipid substrates of ACSL3 that trigger autophagy upon serum lipid depletion, we assessed whether the addition of oleic acid (a lipid substrate of ACSL3) to the cell culture media could rescue serum lipid depletion-induced autophagy. We found that the addition of oleic acid rescued serum lipid depletion-induced autophagy in the presence of ACSL3, but not upon ACSL3 knockdown (Fig. 3F). These data confirm that it is the depletion of extracellularly derived FAs that trigger autophagy and that ACSL3 is important for their activation and retention.

Taken together, our results suggest that the reduction of extracellularly derived lipids, either by serum lipid depletion or ACSL3 inhibition, triggers an increase in autophagic flux, possibly to replenish cancer cells with the necessary lipids.

### Acsl3 deletion enhances autophagy in KPC tumors

In order to assess whether the depletion of extracellularly derived lipids, mediated by ACSL3 suppression, triggers autophagy also in vivo, we generated mice carrying a transgene allowing tamoxifen-dependent expression of *Cre* recombinase under the pancreas-specific *Pdx-1* promoter (*Pdx1-Cre*^*ERT2*^), a *Cre*-activatable Kras^G12D^ allele (*LSL-Kras*^*G12D/+*^), homozygous for a *Cre-*conditional p53 knockout allele (*p53*^*floxflox*^), and either wild type or homozygous for an *Acsl3* knockout allele (*Acsl3*^+/+^ or *Acsl3*^−/−^, respectively), to generate 2 experimental groups: *LSL-Kras*^*G12D/+*^;*p53*^*flox/flox*^;*Pdx1-Cre*^*ERT2*^;*Acsl3*^+/+^ and *LSL-Kras*^*G12D/+*^; *p53*^*flox/flox*^;*Pdx1-Cre*^*ERT2*^;*Acsl3*^−/−^ (hereafter named *KPC*;*Acsl3*^+/+^ and *KPC*;*Acsl3*^−/−^, respectively). Tamoxifen administration to these mice drives the *Cre*-mediated recombination and results in the excision of the *loxP*-flanked stop codon (LSL), thereby leading to conditional expression of *Kras*^*G12D*^ and deletion of *p53* in the pancreas of the mice. The relevant aspect of the KPC mouse model is the spontaneous generation of tumors which faithfully recapitulate the whole spectrum of lesion grades already described in humans [22]. As previously reported, the *KPC;Acsl3*^*−/−*^ mice were born according to the expected Mendelian ratio and without obvious macroscopic defects during development or adult life compared to the *KPC;Acsl3*^*+/+*^ mice [10].

**Fig. 4:**
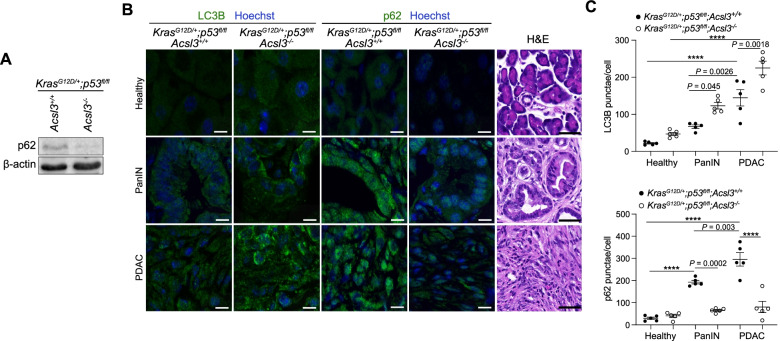
*Acsl3* knockout enhances autophagy in KPC mice. **A** Immunoblot analysis of p62 in pancreatic tumours of *KPC;Acsl3*^+/+^ and *KPC;Acsl3*^−/−^ mice. **B** Representative images of immunofluorescence staining of LC3B (green, left), p62 (green, middle) and nuclei (blue) of pancreatic cancer sections from *KPC;Acsl3*^+/+^ and *KPC;Acsl3*^−/−^ mice; Scale bar 10 μm. Representative images of hematoxylin & eosin (H&E) stained tissue depicting healthy (top), PanINs (middle) and PDAC (bottom) are shown on the right; Scale bar: 50 μm. PanINs: Pancreatic intraepithelial neoplasias. **C** Quantifications of cytoplasmic punctae of LC3B (top) and p62 (bottom) from (B), *n* = 5 mice/group. Graphical data are shown as the mean ± s.d. Statistical analyses were done using two-way ANOVA. **** *P* < 0.0001

Next, we assessed whether *KPC;Acsl3*^*−/−*^ tumors exhibited higher basal autophagy levels compared to *KPC;Acsl3*^*+/+*^. Therefore, we performed immunoblot analysis to assess the degradation of p62 on pancreatic cancer tissue from *KPC;Acsl3*^+/+^ and *KPC;Acsl3*^−/−^ mice. Interestingly, we found reduced p62, indicative of an increased autophagic flux (Fig. 4A). Immunofluorescence (IF) staining of LC3B and p62 on paraffin-embedded tissue from *KPC;Acsl3*^*+/+*^ and *KPC;Acsl3*^*−/−*^ mice followed by computer-assisted quantification revealed a LC3B-positive punctae increase and p62 decrease in PanINs and PDAC tumors of *KPC;Acsl3*^−/−^ mice (Fig. 4B and C). Interestingly, the number of LC3B-positive punctae increased progressively from healthy to PanIN and PDAC lesions (Fig. 4C), confirming previous observations that human PDAC exhibits high levels of basal autophagy [23–25]. Even though not exhaustive in determining flux variations over time, studies involving human patient material with similar LC3B/p62 patterns are interpreted as bona fide evidence of increased autophagy [21, 26].

### Lipid depletion sensitizes PDAC cells to autophagy inhibition in vitro

**Fig. 5:**
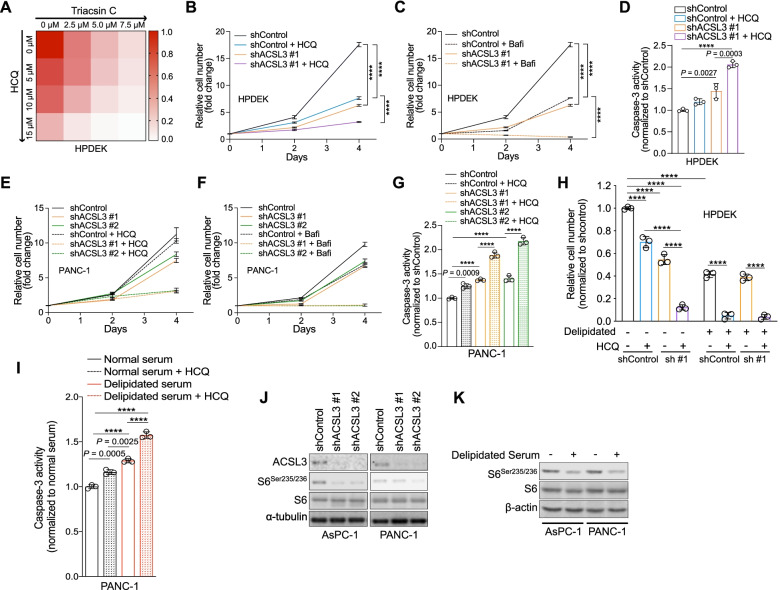
Concomitant extracellular lipid depletion and autophagy targeting strongly affect PDAC cell viability. **A** Heatmap obtained by measurement of HPDEK cell number 96 h upon treatment with incremental combined doses of Triacsin C and HCQ. HCQ: hydroxychloroquine. **B and C** Relative cell number of HPDEK cells transduced with a shRNA against ACSL3 (shACSL3 #1) and treated with 10 μM HCQ (B) or 100 nM bafilomycin (C). HCQ: hydroxychloroquine. Bafi: bafilomycin; *n* = 3. **D** Caspase-3 activity of HPDEK cells treated as in (B). HCQ: hydroxychloroquine; *n* = 3. **E and F** Relative cell number of PANC-1 cells transduced with 2 different shRNAs against ACSL3 and treated with 10 μM HCQ (E) or 100 nM bafilomycin (F). HCQ: hydroxychloroquine. Bafi: bafilomycin; *n* = 3. **G** Caspase-3 activity of PANC-1 cells treated as in (E). HCQ: hydroxychloroquine; *n* = 3. **H** Relative cell number of HPDEK cells transduced with a shRNA against ACSL3 (sh #1: shACSL3 #1) and treated as indicated for 72 h. HCQ: hydroxychloroquine; *n* = 3. **I** Caspase-3 activity of PANC-1 cells treated with media containing normal or delipidated serum for 72 h and/or 10 μM HCQ. HCQ: hydroxychloroquine; *n* = 3. **J and K** Immunoblot analysis for the indicated targets upon ACSL3 knockdown with 2 different shRNAs (J) and upon treatment with media containing delipidated serum for 72 h (K). Graphical data are shown as the mean ± s.d. Statistical analyses were done using one- or two-way ANOVA. **** *P* < 0.0001; n, number of biologically independent samples

Our data link lipid depletion (by serum lipid depletion or ACSL3 knockdown) with reduced proliferation, differential induction of cell death, and concomitant increase in autophagic flux, suggesting that autophagy may play a pro-survival role in this context. Therefore, we aimed to assess whether autophagy inhibition could further enhance the anti-proliferative activity of ACSL3 inhibition. For this purpose, we treated HPDEK cells with increasing concentrations of Triacsin C and/or HCQ and measured cell proliferation. We found that combining ACSL3 with autophagy inhibition cooperated in suppressing cell proliferation in HPDEK cells compared to the single treatments (Fig. 5A).

Next, we employed the HPDEK cells previously transduced with a shRNA against ACSL3 and treated them with HCQ or bafilomycin A1 to inhibit autophagy [27]. We found that ACSL3 knockdown cooperated with HCQ or bafilomycin A1 in suppressing the proliferation of HPDEK cells (Fig. 5B and C). Moreover, the combination of HCQ with ACSL3 knockdown significantly enhanced the caspase-3 activity compared to the single treatments, indicative of enhanced caspase-dependent cell death (Fig. 5D). These results were further confirmed in PANC-1 cells because treatment with HCQ or bafilomycin A1 sensitized PANC-1 cells to the anti-proliferative effect of ACSL3 knockdown (Fig. 5E and F). Notably, the combination of HCQ with ACSL3 knockdown led to a striking induction of caspase-3 activity compared to the single treatments (Fig. 5G). 4-aminoquinolones such as HCQ in addition to inhibiting autophagy have effects also on other cellular processes (i.e. mitochondrial function) [28]. However, since the second autophagy inhibitor, bafilomycin A1, provided similar results we conclude that the cooperative effects that we observed can be ascribed to autophagy inhibition.

Consistent with our data upon ACSL3 knockdown, serum lipid depletion combined with HCQ cooperated in suppressing the proliferation of HPDEK cells (Fig. 5H). Notably, ACSL3 knockdown in cells treated with lipid depleted serum (with or without HCQ treatment) did not enhance further the anti-proliferative effect, confirming that the impact of ACSL3 on cell proliferation is solely dependent on the activation of extracellularly derived lipids (Fig. 5H).

Lastly, serum lipid depletion combined with HCQ treatment enhanced apoptosis compared to the single treatments in both PANC-1 and HPDEK cells (Fig. 5I and Supplementary Fig. 3A).

Taken together, these results suggest that the enhanced autophagy upon extracellular lipid depletion has a pro-survival role, and that concurrent lipid depletion and autophagy inhibition suppresses PDAC proliferation and enhances cell death more effectively than lipid depletion alone.

We next addressed the mechanistic basis of lipid depletion-induced increase in autophagic flux. Similar to serum starvation, lipid depletion is expected to drain cells from essential building blocks. Nutrient starvation is a potent inducer of autophagy through a mechanism involving suppression of the mechanistic target of rapamycin complex 1 (mTORC1) [29]. Therefore, we speculated that extracellular lipid restriction, either by serum lipid depletion or ACSL3 inhibition, induces autophagy by inhibiting the mTORC1 signaling pathway. To test this possibility, we assessed the activation status of a downstream target of mTORC1, S6 kinase. Immunoblot analyses evidenced that upon ACSL3 knockdown (Fig. 5J) or shift to lipid depleted serum (Fig. 5K), the phosphorylated form of S6 appeared reduced, suggesting suppression of the mTORC1 signaling pathway.

### ACSL3 knockdown sensitizes PDAC to autophagy blockade in vivo

**Fig. 6:**
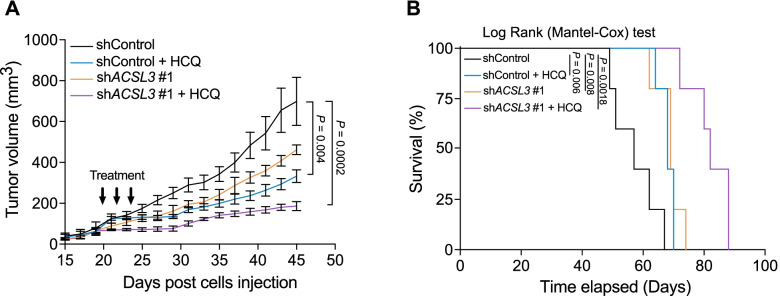
ACSL3 knockdown and autophagy blockade cooperate in suppressing tumor formation in vivo. **A** Tumor burden of PANC-1 xenografts transduced with a shRNA against ACSL3 (shACSL3 #1), before subcutaneous injection into the right flank of the mice and treated with HCQ (60 mg/kg) as indicated by the arrow. Tumors were measured with a caliper every 2 days; *n* = 5 mice/group. **B** Kaplan-Meier graph showing survival of the mice from (A). Death was considered when the tumors reached the endpoint of 1000 mm^3^; *n* = 5 mice/group. Graphical data are shown as the mean ± s.e.m. Statistical analyses were done using one-way ANOVA or log-rank (Mantel–Cox) test (where indicated)

Based on our in vitro findings, we decided to test whether ACSL3 inhibition and autophagy blockade can cooperate in suppressing tumor formation in vivo. To this end, we transduced the human patient-derived PANC-1 cells with a shRNA against ACSL3 and we generated subcutaneous xenograft implants in immunodeficient mice. When the xenografts reached an average size of 100 mm^3^ the mice were treated with HCQ and the size of the xenograft tumors was measured with a caliper over time. Notably, the combination treatment was more effective in suppressing tumor progression compared to the vehicle control or single treatments (Fig. 6A). Moreover, the combination therapy significantly increased the survival of mice compared to the single treatments (Fig. 6B).

Taken together, our results indicate that the combination of autophagy and ACSL3 inhibition in vivo greatly improves the efficacy and survival outcome of the single treatments providing the rationale for future implementation of this combination strategy.

## Discussion

The metabolic reprogramming of cancer cells although contributing to tumor progression introduces cancer-specific metabolic liabilities that can be exploited for therapy. However, strategies to target metabolic pathways in cancer have not fully translated into compelling clinical results due to the flexibility of the cancer metabolic network, providing alternative metabolic routes. Here we found that in PDAC cells the restriction of extracellular lipids, either by serum lipid depletion or ACSL3 suppression in vitro and in vivo, is compensated by increased autophagic flux. Indeed, combination of lipid depletion with autophagy inhibitors dramatically suppressed the proliferation of PDAC cells and increased apoptosis compared to the single interventions. Therefore, our work unravels a central role of extracellular lipid supply in ensuring FA provision in cancer cells, unmasking a previously unappreciated metabolic vulnerability of PDAC cells.

The differential induction of cell death in pancreatic cancer cell lines upon extracellular lipid restriction, either by ACSL3 knockdown or serum lipid depletion, underscores the heterogeneity of human pancreatic tumors. However, the extracellular lipid restriction consistently increased autophagy in all cell lines tested and sensitized them to autophagy inhibitors, indicating a common metabolic vulnerability that could be exploited for cancer therapy.

Our data show that inhibition of autophagy increases the content of lipid droplets in PDAC cells. Thus, autophagy can directly contribute to the utilization of cellular lipid stores, a metabolic process that can become indispensable when extracellularly derived FAs cannot be utilized. Future studies are warranted to understand which FAs are recycled by autophagy in PDAC cells upon extracellular FA depletion.

As previously reported for human PDAC [23, 25], we observed that mouse PDAC exhibits high levels of basal autophagy compared to healthy tissue and PanIN lesions (Fig. 4). Given the high steady-state levels of autophagy in human PDAC, it was expected that inhibition of autophagy could suppress PDAC growth. However, this was not the case as ongoing clinical trials have observed limited to no efficacy of HCQ, an orally administered FDA-approved drug [30]. Our data show that extracellular lipid suppression rendered PDAC cells dependent on autophagy. Interestingly, concurrent extracellular lipid suppression cooperated in enhancing hydroxychloroquine-mediated growth inhibition in preclinical models, suggesting that ACSL3 inhibition or dietary intervention could improve the efficacy of autophagy inhibitors in PDAC patients.

Perturbations to dietary fat compositions, in principle, can change FA levels in circulation, which in turn can influence FA levels in the tumor microenvironment. Thus, diet-induced changes in nutrient availability could affect cancer cell proliferation, thereby altering tumor growth, progression, and response to therapy. Although we do not know how dietary fat factors modulate the systemic availability of lipids that are available for use by cancer cells within a tumor, it is interesting to note that low-fat dietary intervention is associated with reduced pancreatic cancer incidence in women who were overweight or obese (clinical trial identifier: NCT00000611) [31]. Given that the incidence and prognosis of pancreatic cancers are highly affected by high levels of circulating unsaturated FAs [32, 33], these results suggest that low-fat diet may be a promising strategy to treat patients with pancreatic cancer and use in combination therapies with HCQ. Future studies are warranted to understand whether different dietary fat compositions can directly affect the availability of FAs in PDAC tumors.

## Conclusions

In this study, we aimed to understand the role of extracellularly derived lipids on the proliferation and survival of pancreatic cancer cells. We found that PDAC cells survive extracellularly derived lipids depletion or ACSL3 suppression by increasing autophagy. Concomitant extracellularly derived lipid depletion and autophagy inhibition suppressed tumor progression, suggesting that this may be an effective combination strategy to target PDAC tumors. Therefore, we have exposed a new metabolic liability of PDAC tumors that could be exploited for therapeutic purposes.

## Supplementary Information

**Additional file 1.**

## Data Availability

Further information and requests for resources and reagents should be directed to and will be fulfilled by the corresponding author, Georgia Konstantinidou, Institute of Pharmacology, University of Bern, 3010 Bern, Switzerland (georgia.konstantinidou@pki.unibe.ch). Requests will be handled according to the University of Bern policies regarding MTA and related matters.

## Abbreviations

PDAC: Pancreatic ductal adenocarcinomas
PanIN: Pancreatic intraepithelial neoplasia
ACSL3: Acyl-CoA Synthetase long chain 3
FAs: Fatty acids
KRAS: Kirsten rat sarcoma virus
HCQ: Hydroxychloroquine
mTORC1: Mechanistic target of rapamycin complex 1
KPC: *Kras* ^*G12D/+*^;*p53*^*flox/flox*^;*Pdx1-Cre*^*ERT2*^
LC3B: Microtubule-associated protein 1 light chain 3 beta
shRNA: Short hairpin RNA
ANN/PI: Annexin V/propidium iodide

## References

[1] Viale A, Pettazzoni P, Lyssiotis CA, Ying H, Sanchez N, Marchesini M (2014). Oncogene ablation-resistant pancreatic cancer cells depend on mitochondrial function. Nature.

[2] Siegel RL, Miller KD, Fuchs HE, Jemal A (2021). Cancer statistics, 2021. CA Cancer J Clin.

[3] Neoptolemos JP, Kleeff J, Michl P, Costello E, Greenhalf W, Palmer DH (2018). Therapeutic developments in pancreatic cancer: current and future perspectives. Nat Rev Gastroenterol Hepatol.

[4] Leroux C, Konstantinidou G. Targeted therapies for pancreatic Cancer: overview of current treatments and new opportunities for personalized oncology. Cancers (Basel). 2021;13(4). 10.3390/cancers13040799 PMC7918504 33672917

[5] Pupo E, Avanzato D, Middonti E, Bussolino F, Lanzetti L (2019). KRAS-driven metabolic rewiring reveals novel actionable targets in Cancer. Front Oncol.

[6] Kamphorst JJ, Cross JR, Fan J, de Stanchina E, Mathew R, White EP (2013). Hypoxic and Ras-transformed cells support growth by scavenging unsaturated fatty acids from lysophospholipids. Proc Natl Acad Sci U S A.

[7] Kamp F, Hamilton JA (2006). How fatty acids of different chain length enter and leave cells by free diffusion. Prostaglandins Leukot Essent Fatty Acids.

[8] Rossi Sebastiano M, Konstantinidou G. Targeting long chain acyl-CoA Synthetases for Cancer therapy. Int J Mol Sci. 2019;20(15). 10.3390/ijms20153624 PMC6696099 31344914

[9] Padanad MS, Konstantinidou G, Venkateswaran N, Melegari M, Rindhe S, Mitsche M (2016). Fatty acid oxidation mediated by acyl-CoA Synthetase long chain 3 is required for mutant KRAS lung tumorigenesis. Cell Rep.

[10] Rossi Sebastiano M, Pozzato C, Saliakoura M, Yang Z, Peng RW, Galie M, et al. ACSL3-PAI-1 signaling axis mediates tumor-stroma cross-talk promoting pancreatic cancer progression. Sci Adv. 2020;6(44). 10.1126/sciadv.abb9200 PMC7608806 33127675

[11] Saliakoura M, Reynoso-Moreno I, Pozzato C, Rossi Sebastiano M, Galie M, Gertsch J (2020). The ACSL3-LPIAT1 signaling drives prostaglandin synthesis in non-small cell lung cancer. Oncogene.

[12] Tsang YH, Dogruluk T, Tedeschi PM, Wardwell-Ozgo J, Lu H, Espitia M (2016). Functional annotation of rare gene aberration drivers of pancreatic cancer. Nat Commun.

[13] Saliakoura M, Rossi Sebastiano M, Pozzato C, Heidel FH, Schnoder TM, Savic Prince S (2020). PLCgamma1 suppression promotes the adaptation of KRAS-mutant lung adenocarcinomas to hypoxia. Nat Cell Biol.

[14] Majka SM, Miller HL, Helm KM, Acosta AS, Childs CR, Kong R (2014). Analysis and isolation of adipocytes by flow cytometry. Methods Enzymol.

[15] Qiu B, Simon MC (2016). BODIPY 493/503 staining of neutral lipid droplets for microscopy and quantification by flow Cytometry. Bio Protoc.

[16] Furukawa T, Duguid WP, Rosenberg L, Viallet J, Galloway DA, Tsao MS (1996). Long-term culture and immortalization of epithelial cells from normal adult human pancreatic ducts transfected by the E6E7 gene of human papilloma virus 16. Am J Pathol.

[17] Singh R, Kaushik S, Wang Y, Xiang Y, Novak I, Komatsu M (2009). Autophagy regulates lipid metabolism. Nature.

[18] Bosc C, Broin N, Fanjul M, Saland E, Farge T, Courdy C (2020). Autophagy regulates fatty acid availability for oxidative phosphorylation through mitochondria-endoplasmic reticulum contact sites. Nat Commun.

[19] Tschan MP, Fischer KM, Fung VS, Pirnia F, Borner MM, Fey MF (2003). Alternative splicing of the human cyclin D-binding Myb-like protein (hDMP1) yields a truncated protein isoform that alters macrophage differentiation patterns. J Biol Chem.

[20] Rizzi M, Tschan MP, Britschgi C, Britschgi A, Hugli B, Grob TJ (2007). The death-associated protein kinase 2 is up-regulated during normal myeloid differentiation and enhances neutrophil maturation in myeloid leukemic cells. J Leukoc Biol.

[21] Klionsky DJ, Abdelmohsen K, Abe A, Abedin MJ, Abeliovich H, Acevedo Arozena A (2016). Guidelines for the use and interpretation of assays for monitoring autophagy (3rd edition). Autophagy.

[22] Guerra C, Barbacid M (2013). Genetically engineered mouse models of pancreatic adenocarcinoma. Mol Oncol.

[23] Yang S, Wang X, Contino G, Liesa M, Sahin E, Ying H (2011). Pancreatic cancers require autophagy for tumor growth. Genes Dev.

[24] Yang A, Rajeshkumar NV, Wang X, Yabuuchi S, Alexander BM, Chu GC (2014). Autophagy is critical for pancreatic tumor growth and progression in tumors with p53 alterations. Cancer Discov.

[25] Guo JY, Chen HY, Mathew R, Fan J, Strohecker AM, Karsli-Uzunbas G (2011). Activated Ras requires autophagy to maintain oxidative metabolism and tumorigenesis. Genes Dev.

[26] Niklaus M, Adams O, Berezowska S, Zlobec I, Graber F, Slotta-Huspenina J (2017). Expression analysis of LC3B and p62 indicates intact activated autophagy is associated with an unfavorable prognosis in colon cancer. Oncotarget.

[27] Yamamoto A, Tagawa Y, Yoshimori T, Moriyama Y, Masaki R, Tashiro Y (1998). Bafilomycin A1 prevents maturation of autophagic vacuoles by inhibiting fusion between autophagosomes and lysosomes in rat hepatoma cell line, H-4-II-E cells. Cell Struct Funct.

[28] Boya P, Gonzalez-Polo RA, Poncet D, Andreau K, Vieira HL, Roumier T (2003). Mitochondrial membrane permeabilization is a critical step of lysosome-initiated apoptosis induced by hydroxychloroquine. Oncogene.

[29] Jung CH, Ro SH, Cao J, Otto NM, Kim DH (2010). mTOR regulation of autophagy. FEBS Lett.

[30] Wolpin BM, Rubinson DA, Wang X, Chan JA, Cleary JM, Enzinger PC (2014). Phase II and pharmacodynamic study of autophagy inhibition using hydroxychloroquine in patients with metastatic pancreatic adenocarcinoma. Oncologist.

[31] Jiao L, Chen L, White DL, Tinker L, Chlebowski RT, Van Horn LV, et al. Low-fat dietary pattern and pancreatic Cancer risk in the Women's Health Initiative dietary modification randomized controlled trial. J Natl Cancer Inst. 2018;110(1). 10.1093/jnci/djx117 PMC6059156 28922784

[32] Nkondjock A, Krewski D, Johnson KC, Ghadirian P (2005). Canadian Cancer registries epidemiology research G. specific fatty acid intake and the risk of pancreatic cancer in Canada. Br J Cancer.

[33] Thiebaut AC, Jiao L, Silverman DT, Cross AJ, Thompson FE, Subar AF (2009). Dietary fatty acids and pancreatic cancer in the NIH-AARP diet and health study. J Natl Cancer Inst.

