# Supplementary material for: Restriction of extracellular lipids renders pancreatic cancer dependent on autophagy
Source: J Exp Clin Cancer Res. 2022 Jan 8;41:16. doi: 10.1186/s13046-021-02231-y (PMC8742413; doi:10.1186/s13046-021-02231-y)
Supplement: Supplementary file 1 — Additional file 1. [file 13046_2021_2231_MOESM1_ESM.pdf]

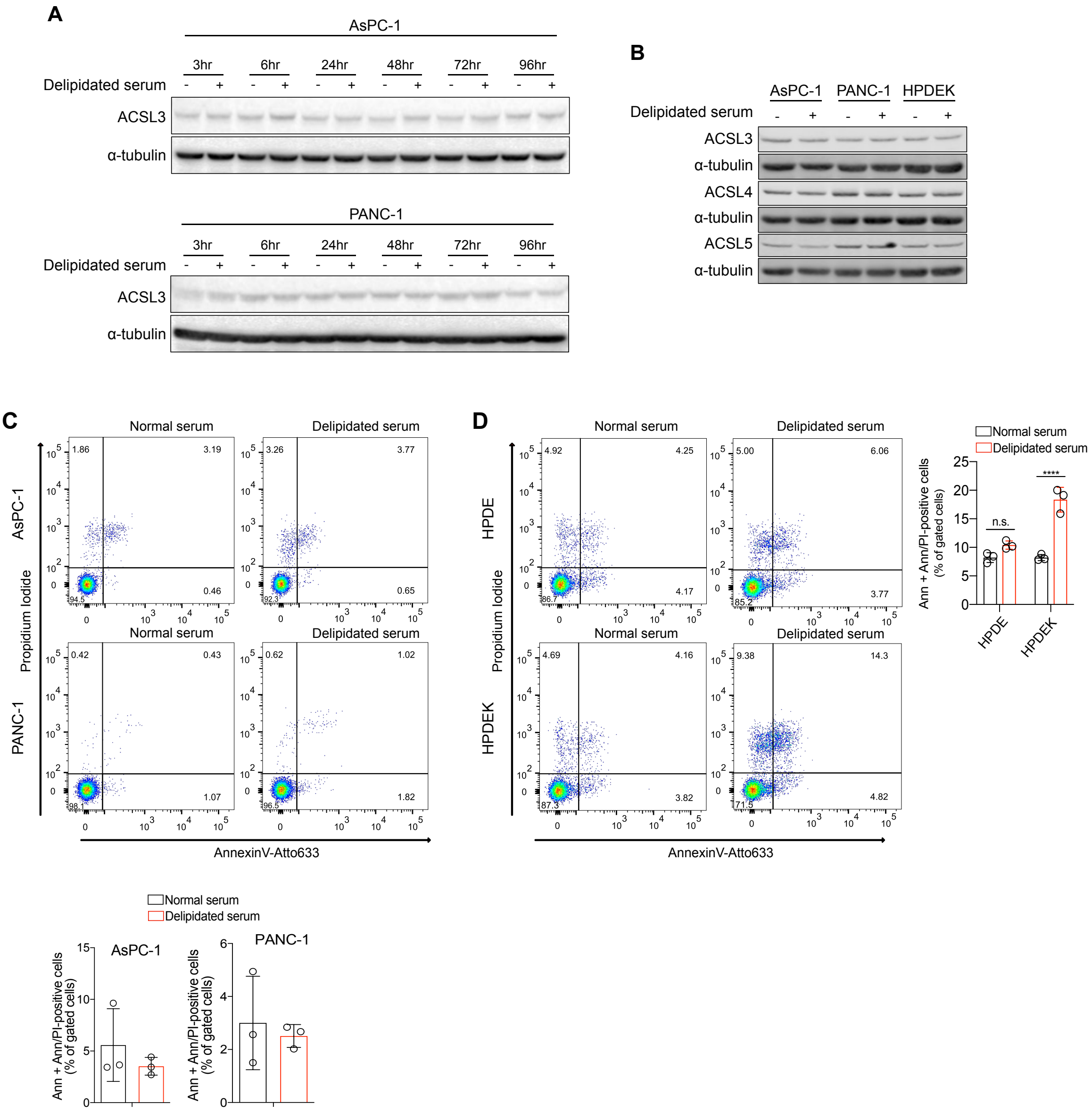

Supplementary Figure 1. Serum lipid depletion induces apoptotic cell death in HPDEK cells, but not in PDAC cell lines or HPDE cells.

(A) Immunoblot showing ACSL3 protein levels of AsPC-1 (top) or PANC-1 (bottom) PDAC cell lines upon treatment with media containing normal or lipid depleted serum for the indicated time.

(B) Immunoblot showing ACSL3, ACSL4 and ACSL5 protein levels of the indicated PDAC cell lines upon treatment with media containing normal or lipid depleted serum for 72h.

(C) Representative panels of Ann–Atto633/PI flow cytometry analysis (top) and relative quantification of Ann + Ann/PI-positive AsPC-1 and PANC-1 cells (bottom) treated with media containing normal or delipidated serum for 72h; n = 3.

(D) Representative panels of Ann–Atto633/PI flow cytometry analysis (left) and relative quantification of Ann + Ann/PI-positive HPDE and HPDEK cells (right) treated as in (C); n = 3.

Graphical data are shown as the mean ± s.d. Statistical analyses were done using two-tailed unpaired Student's t-test or one-way ANOVA; n, number of biologically independent samples.

Supplementary Figure 2 Saliakoura M. et al.

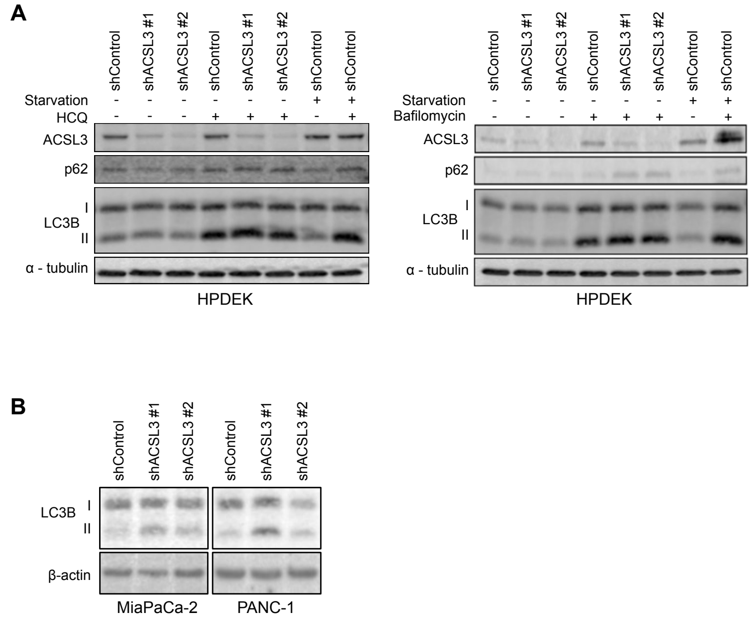

Supplementary Figure 2. ACSL3 knockdown induces autophagy in pancreatic cancer cells.

(A) Immunoblot for the indicated targets of HPDEK cells transduced with 2 different shRNAs against ACSL3 and treated with 10μM HCQ (left) or 100nM bafilomycin (right) for 4h. HCQ: hydroxychloroquine; Serum starvation was used as a positive control for autophagy induction.

(B) Immunoblot for LC3B in the indicated cell lines transduced as in (A).

Supplementary Figure 3 Saliakoura M. et al.

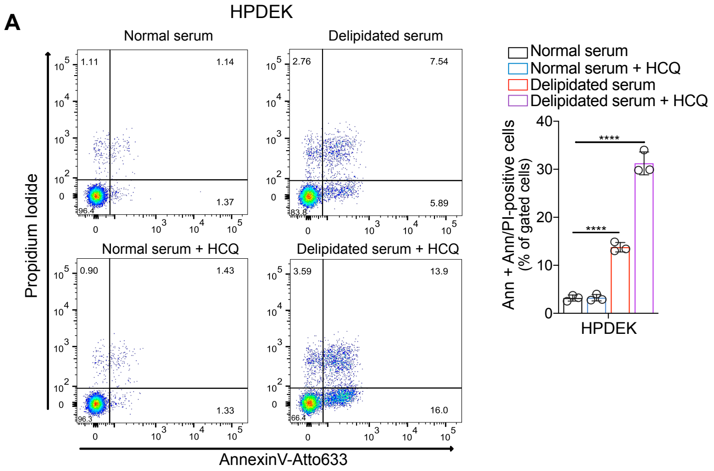

Supplementary Figure 3. Autophagy inhibition enhances serum lipid depletion-induced apoptosis in HPDEK cells.

(A) Representative panels of Ann–Atto633/PI flow cytometry analysis (left) and relative quantification of Ann + Ann/PI-positive HPDEK cells (right) treated as indicated; n = 3. HCQ: hydroxychloroquine.

Graphical data are shown as the mean ± s.d. Statistical analyses were done using one-way ANOVA; n, number of biologically independent samples.
